# MicroBubble activated acoustic cell sorting

**DOI:** 10.1007/s10544-017-0157-4

**Published:** 2017-04-03

**Authors:** M. A. Faridi, H. Ramachandraiah, I. Iranmanesh, D. Grishenkov, M. Wiklund, A. Russom

**Affiliations:** 1 Division of Proteomics and Nanobiotechnology, Science for Life Laboratory, KTH Royal Institute of Technology, Stockholm, Sweden; 2 Deptartment of Applied Physics, School of Engineering Science, KTH Royal Institute of Technology, Stockholm, Sweden; 3 Department of Medical Engineering, School of Technology and Health, KTH Royal Institute of Technology, Stockholm, Sweden

**Keywords:** Cell sorting, Acoustophoresis, Microbubble, Contrast agent, Microfluidic separation

## Abstract

Acoustophoresis, the ability to acoustically manipulate particles and cells inside a microfluidic channel, is a critical enabling technology for cell-sorting applications. However, one of the major impediments for routine use of acoustophoresis at clinical laboratory has been the reliance on the inherent physical properties of cells for separation. Here, we present a microfluidic-based microBubble-Activated Acoustic Cell Sorting (BAACS) method that rely on the specific binding of target cells to microbubbles conjugated with specific antibodies on their surface for continuous cell separation using ultrasonic standing wave. In acoustophoresis, cells being positive acoustic contrast particles migrate to pressure nodes. On the contrary, air-filled polymer-shelled microbubbles being strong negative acoustic contrast particles migrate to pressure antinodes and can be used to selectively migrate target cells. As a proof of principle, we demonstrate the separation of cancer cell line in a suspension with better than 75% efficiency. Moreover, 100% of the microbubble-cell conjugates migrated to the anti-node. Hence a better upstream affinity-capture has the potential to provide higher sorting efficiency. The BAACS technique expands the acoustic cell manipulation possibilities and offers cell-sorting solutions suited for applications at point of care.

**Electronic supplementary material:**

The online version of this article (doi:10.1007/s10544-017-0157-4) contains supplementary material, which is available to authorized users.

## Introduction

The isolation of pure cell population from complex biological sample is a pre-requisition for routine diagnostics. Selective cell sorting is conventionally performed in fluorescent activated cell sorters (FACS) and magnetic-activated cell sorters (MACS) that use fluorescent antibody and antibody coated magnetic beads conjugated to the cells, respectively. These systems are costly and are often limited to equipped laboratories. Fuelled by the need towards miniaturization for clinical point of care diagnostics, a wide range of microfluidic cell-sorting devices has emerged in recent years. Similar to macroscale methods, these microfluidic devices separate cells based on their physical, chemical and functional properties. These cell isolation methods can be roughly divided into active and passive systems. Cell separation can be done either by using hydrodynamic forces acting on the particles in a microfluidic channel based on the geometrical design of the channel or flow regimes (passive particle manipulation) (Gossett et al. 2010; Weigl et al. 2000) using deterministic lateral displacement (DLD) (Huang 2004) and inertial microfluidics (Di Carlo 2009; Russom et al. 2009) or by applying external forces on the particles flowing inside a microfluidic channel (active particle manipulation) such as electrophoresis (Dolník et al. 2000), acoustophoresis (Lenshof and Laurell 2010) and magnetophoresis (Pamme and Manz 2004).

Acoustofluidics (Laurell and Lenshof 2014) is a branch of microfluidics dealing with ultrasound waves that are coupled to microfluidic channel creating acoustic forces in different forms (mainly from bulk or surface waves). This allows manipulation of particles inside the channel referred to as acoustophoresis (Bruus et al. 2011) for different purposes. The advantages using this method are robustness, gentleness and especially for bulk acoustic systems the simplicity of the device, which allows; separation (Dykes et al. 2011; Petersson et al. 2007; Thévoz et al. 2010), enrichment (Augustsson et al. 2012), up-concentration (Hammarström et al. 2012) or washing (Hawkes et al. 2004) of the samples inside the microfluidic chip.

Despite all the advantages offered by acoustophoresis, the acoustic radiation force is highly dependent on the size of the particles, which for some applications can be a drawback. For example, it is difficult to distinguish between bioparticles of the same size but with different biological characteristics, when it comes to sorting of the sample of interest for further analysis or performing specific bioassays having a mixed sample with narrow size distribution. Recently, Augustsson et al. used a method called iso-acoustic particle manipulation (Augustsson et al. 2016). The technique allows for separating cells of the same size by introducing an acoustic contrast gradient of the medium, since the acoustophoretic contrast factor of cell is calculated relative to the suspending medium’s density and compressibility. In the work by Augustsson et al., the medium density was altered such that the suspend cell would behave as positive, negative or neutral acoustophoretic particles. The gradient within the channel was created such that the cells depending on their relative contrast profile would laterally migrate in the standing wave to reach an equilibrium position where the primary radiation force and acoustic contrast vanishes in the iso-acoustic point. The technique is very powerful, but creating the optimized flow for diffusion based gradient generation need complex fluidic setups. Moreover the use of Iodixanol as a medium contrast altering agent might induce toxic effect to some cells, e.g. human renal cells (HEK293) (Romano et al. 2008). Another acoustophoretic method for size-independent cell separation is to use biofunctionalized negative acoustic contract particles (NACPs). This method has been demonstrated by the use of silicone-based elastomeric NACPs for the separation of elastomeric particles from polystyrene particles (Johnson et al. 2013) and from cells (Cushing et al. 2013; Shields et al. 2014). The method based on elastomeric particles has great potential, although the separation efficiency is not fully clear under continuous-flow conditions.

In this paper we use antibody-functionalized microbubble (MB) as NACPs for assisting size-independent particle and cell separation in a microfluidic channel. These NACPs are highly interesting for acoustophoretic separations since they have a strong negative acoustic contrast factor several orders of magnitude larger than solid particles including elastomers (Kothapalli et al. 2016). We study the separation of HCT 116 colon carcinoma cell lines by the use of air-filled polymer-shelled MBs functionalized with EpCam antibodies. We report selective migration of MB-cell complexes to pressure antinode in no flow condition. Finally we demonstrate selective sorting of MB-cell complexes with efficiency of 75% at the flow rate 180 μl/h.

## Theoretical background

Acoustophoresis based separation of cells in microfluidic channels employs the generation of ultrasonic sound wave field within the channel, where primary radiation force (*F*_PRF_) acts on the particles with the magnitude corresponding to their size and direction corresponding to their relative density and compressibility to medium (Bruus 2012).

1 $$ {\boldsymbol{F}}_{\mathbf{PRF}}=4/3 \times {\boldsymbol{\pi}\ \boldsymbol{a}}^3{\boldsymbol{E}}_{\mathbf{ac}}\boldsymbol{k}\boldsymbol{\sin}\left(2\boldsymbol{kz}\right)\ \boldsymbol{\phi} \left(\boldsymbol{\rho}, \boldsymbol{\kappa} \right) $$

In Eq. (1) *a* is the radius of the particle, *k* is the wave number (2π/λ), *E*_ac_ is the time-averaged acoustic energy density and *ϕ*(*ρ*, *κ*) is the acoustophoretic contrast factor (ACF) that can be expressed as follows:

2 $$ \boldsymbol{\phi} \left(\boldsymbol{\rho}, \boldsymbol{\kappa} \right)=\left(5{\boldsymbol{\rho}}_{\mathbf{p}} - 2{\boldsymbol{\rho}}_{\mathbf{o}}\right)/\left(2{\boldsymbol{\rho}}_{\mathbf{p}} + {\boldsymbol{\rho}}_{\mathbf{o}}\right)-{\boldsymbol{\kappa}}_{\mathbf{p}}{/\boldsymbol{\kappa}}_{\mathbf{o}} $$

In Eq. (2) the *ρ*_p_ and *ρ*_o_ are density of the particle and medium, respectively, while *κ*_p_ and *κ*_o_ are the particle and medium compressibility (Bruus 2012).

The particles that are affected by the radiation force migrate to either pressure node or pressure antinode depending upon their relative density and compressibility (Barnkob et al. 2012). The particle with density higher than the suspending medium liquid and compressibility lower than that are positive acoustic contrast particles (PACP) and they migrate to pressure nodes (Bruus 2012). Since cell are PACP they can be separated based on their size and contrast factor towards the nodes, and this phenomena is exploited for cell separation in various applications (Dykes et al. 2011; Hawkes and Coakley 2001; Laurell et al. 2007; Nilsson et al. 2004; Thévoz et al. 2010). On the other hand the particles with density lower than the suspending medium and compressibility higher than that are negative acoustic contrast factor particles (NACP) and they migrate towards pressure antinodes (Bruus 2012). This phenomena has been employed in three different ways: (a) separation of NACP from PACP; lipid (NACP) separation from erythrocyte (PACP) in no flow and flow conditions (Jönsson et al. 2004; Petersson et al. 2005; Petersson et al. 2004) as well as applications like lipid separation for milk processing (Grenvall et al. 2009); (b) altering the relative ratio of density of particle to medium by chemically changing the medium’s density to achieve separation (Augustsson et al. 2016; Petersson et al. 2007) in flow-through condition; (c) selectively attaching PACP with NACP to migrate the resulting complex towards antinodes (Johnson et al. 2013) in no-flow condition.

## Materials and methods

### Synthesis of microbubbles (MBs)

We have utilized poly(vinyl alcohol), PVA, based microbubbles as NACP. The MBs are obtained by foaming a water solution of PVA previously oxidized with sodium metaperiodate. Poly(vinyl alcohol) and sodium metaperiodate are products of Sigma Aldrich. The PVA chains are cross-linked during reaction occurring at the water/air interface (Cavalieri et al. 2005). Resulting MBs have an air-filled core stabilized by a highly hydrated polymer shells having an average diameter of 3.56 ± 1.08 μm with a shell thickness of about 200 nm. These MBs were used for initial characterization using bright field microscopy.

### Cell-MBs conjugation

For affinity-based experiments, the MBs were labeled with rhodamine fluorescent dye and functionalized with streptavidin by Surflay Nanotech Gmbh. HCT 116 colon cancer cells were cultured until 85% confluence in McCoy’s 5a media along with with 2 mM Glutamine and 10% Fetal Bovine Serum and incubated at 37 °C under 5% CO_2_. They were harvested using trypsin-EDTA (Life technologies) for 2 min followed by mechanical dissociation by pipetting for single cell suspension. Cells were passaged every 2 to 3 days. HCT 116 colon cancer cell lines were stained using Calcein-AM (Sigma-Aldrich). For the affinity capture of cells, the streptavidin coated MBs were first incubated with biotinylated anti-EpCAM antibodies for 15 min at room temperature, centrifuged and the pellet was washed thrice. The cells were then incubated with the MBs for 15 min.

### Experimental setup

The setup consisted of single inlet single outlet, glass- silicone microfluidic chip (GeSim GmbH, Dresden, Germany) of dimension 110 × 535 μm (height × width). For acoustophoretic manipulation the lead zirconate titanium (PZT) transducer with fundamental driving frequency of 2.8 MHz is mounted on the chip by water-soluble glue (Tensive conductive adhesive gel by Perker Labs Inc. USA). The PZT was driven with continuous sinusoidal wave by a function generator (AFG 3022, Tektronix Inc., USA). The MBs solution together with either 10 μm fluorescence particles and/or MBs conjugated with cancer cells were introduced into the microchannel using a syringe pump (Harvard apparatus PHD 2000, Harvard Apparatus, USA) and the images were acquired using an inverted fluorescent microscope. Obtained images were analyzed using the software ImageJ.

## Results and discussion

**Fig. 1:**
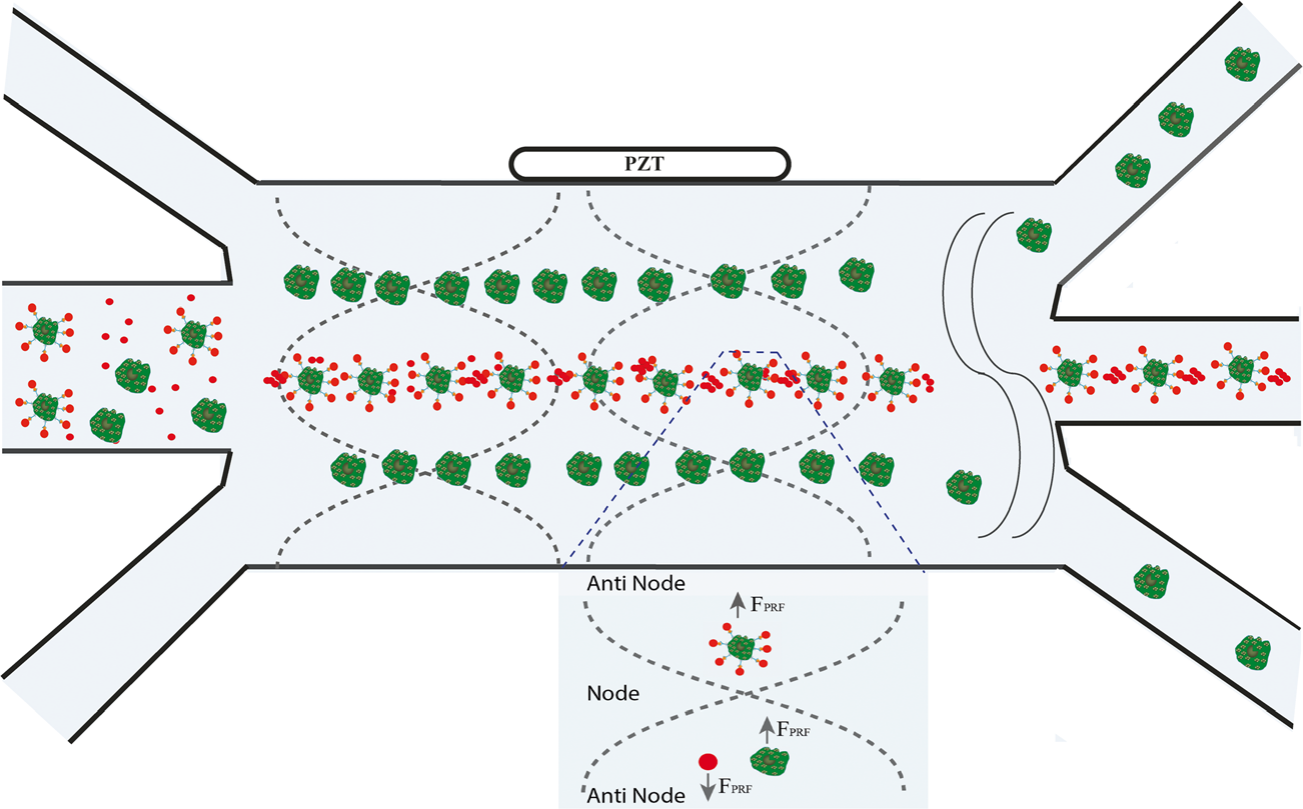
Schematic illustration of the microbubble-activated acoustic cell sorting (BAACS) using immunoaffinity cell capture with antibody-coated MBs. Target cells, affinity conjugated to MBs, migrate towards the antinode while none-target cells migrate towards the nodes and can be separated

The principle of the method we term “microBubble-activated Acoustophoretic Cell Sorting (BAACS)” is shown in Fig. 1. Briefly, microbubbles coated with specific antibody are mixed with the target cells in a suspension. After mixing, target cells attached with microbubbles are pumped through the microfluidic channel under a constant acoustic standing wave and strong negative ACF of MBs in the acoustic forces drag the attached cells to anti-nodes while non-target cells are migrated to node for separation. In the following sections, we will first briefly describe and discuss the focusing phenomena of the MBs to antinodes in stationary and flow through microfluidic channels, after which we present our data on sorting and separating affinity-captured cancer cells.

### On chip characterization of MBs under acoustics

**Fig. 2:**
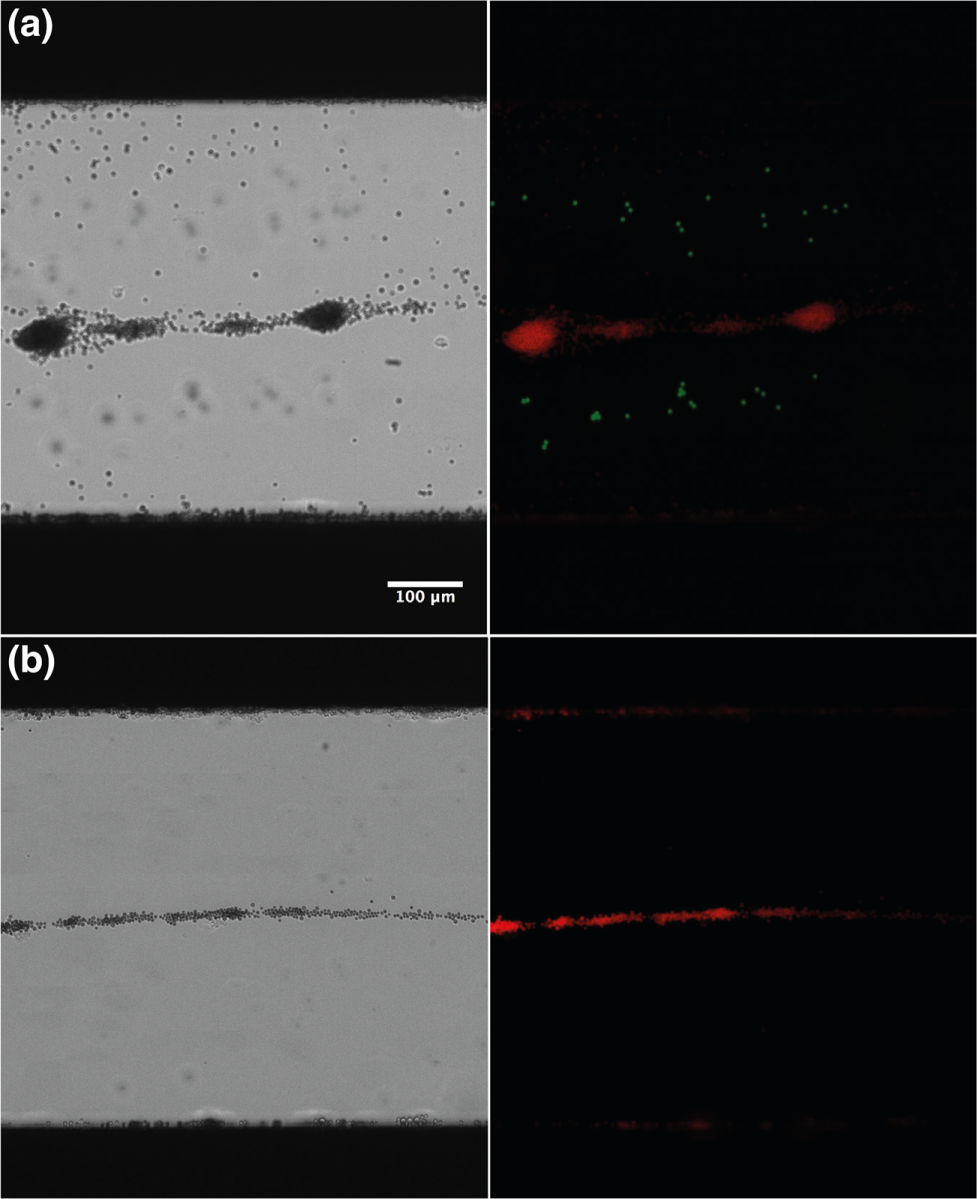
Trapping of polystyrene particles and MB at stationary and flow-through conditions. **a** Bright field (*left*) and fluorescent image (*right*) of streptavidin coated rhodamine labeled MB (*red*) trapping at antinodes (0, λ, λ/2) mostly at middle antinode and polystryrene particles (*green*) trapping at nodes (λ/4, 3λ/4),in standing waves at 140 kPa. **b** The bright field (*right*) and fluorescent image (*left*) of MBs flowing in standing waves through antinodes at the center

Following the protocol reported previously by Kothapalli el al. (Kothapalli et al. 2016) we studied the mixture of polystyrene particles and MBs using bright field microscopy, first at no-flow condition and then at flow through condition where flow rate was varied from 0 to 80 μl/min. As can be seen in Fig. 2a, at no-flow condition, the MBs being higher compressible and lower in density than surrounding medium (PBS) moved to antinodes (0, λ/2 and λ) and trapped there, while the polystyrene microparticles aligned with the pressure node planes and formed two lines at λ/4 and 3λ/4 i.e., at positions 0.25 times diameter away from the walls of microchannel. The polystyrene particles were almost unaffected at the lower pressure below 100 kPa (5 Vpp voltage through PZT) however they migrated to the pressure nodes at higher pressure. At flow-through experiments we observed that bubbles keep accumulating and flowing at the middle antinode position in the channel at the pressure under 120 kPa at all values of the flow rates up to 80 μl/min. As can be seen in Fig. 2b, the MBs flow through antinodes under the acoustic standing wave field. By optimizing the flow condition, such that the drag forces in flow condition is higher than the lateral component of primary radiation forces, most of the MBs will flow through the middle antinode (λ), as can be seen in Supplementary video 1. When the radiation pressure amplitude is high and flow rate is low, the bubbles will tend to accumulate in middle antinode and migrate to antinodes along the walls of channel and, as can be seen in Supplementary video 2. To keep the bubbles flowing along the middle anti-node position, we optimized the flow condition by adjusting flow rate and the radiation pressure (adjusting voltage Vpp) as can be seen in Supplementary video 1.

### Functionalized MB-cell sorting

**Fig. 3:**
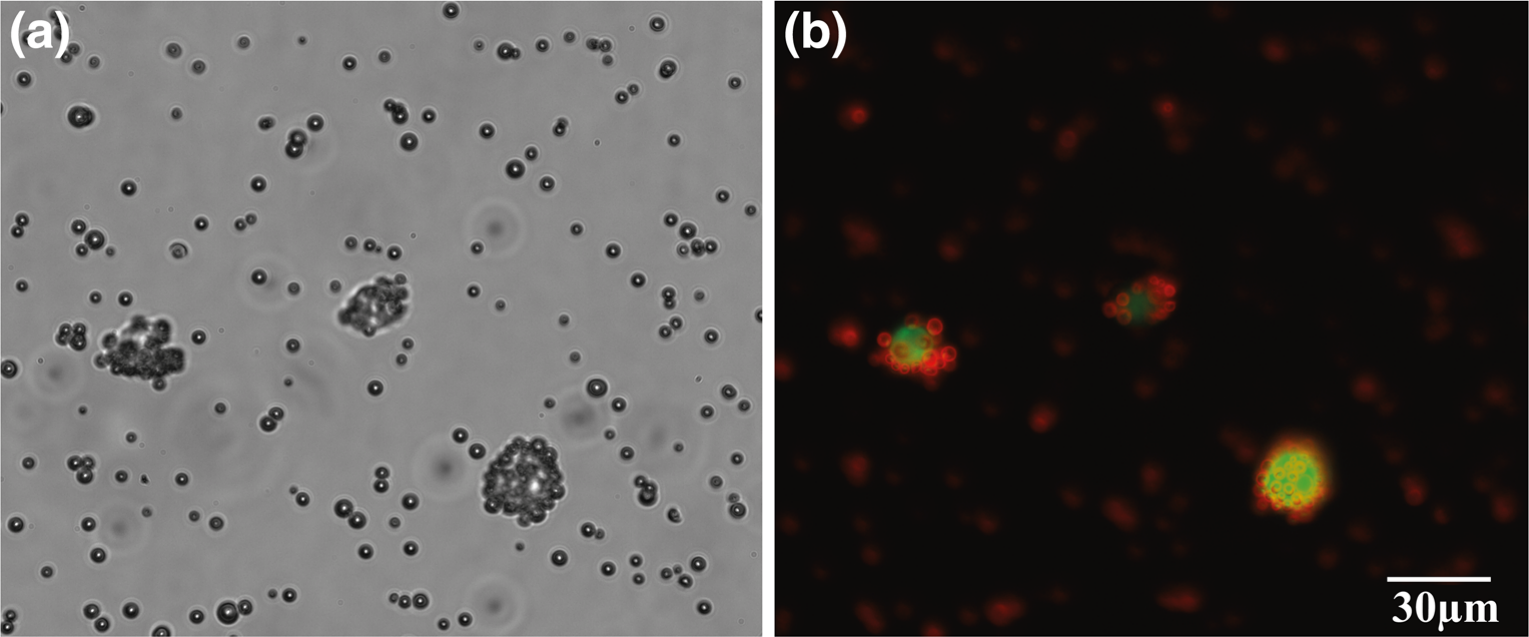
Cell–MBs binding. **a** Bright field and **b** fluorescent image where the MBs are labeled red and the cancer cell lines green

**Fig. 4:**
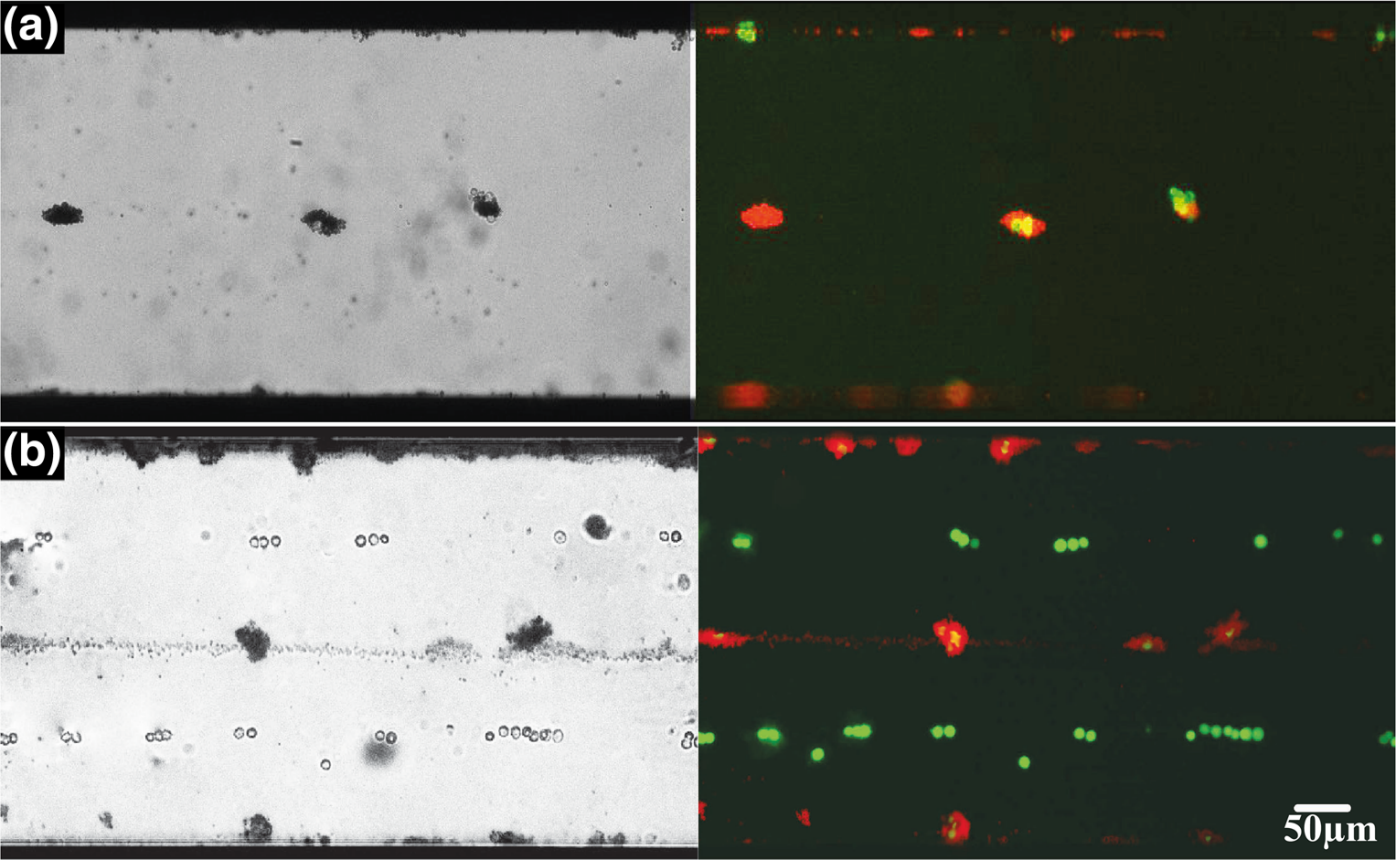
Acoustic based sorting in stationary condition. **a** The MBs-cell complex are at antinodes under acoustic field, and **b** the mixture of cells (*green*) and MBs-cell complex (*red-green*) where cells are at nodes and MBs-cell complex at antinodes

The MBs were first conjugated with streptavidine using standard silane chemistry. This is then followed by incubation with biotinylated antibody towards a target cell. Here, we have targeted epithelial cell adhesion molecule (EpCAM) as a cell surface marker for isolation of circulating tumor cells. Figure 3 shows MB-cancer cell line after the affinity capturing. Initially, we exposed the MB-cell complex to standing wave under no-flow condition. Figure 4a shows bright field (left) and fluorescent (right) image of acoustic positioning of the MBs-cell complex to antinodes in no flow condition. Next, we added a mixture of MBs-cell complex and unbounded cells into the chip and the MBs-cell complexes were sorted to antinodes (center and along the walls) and the unbounded cells positioned at node i.e., at positions 0.25 times diameter away from the walls of microchannel (Fig. 4b).

**Fig. 5:**
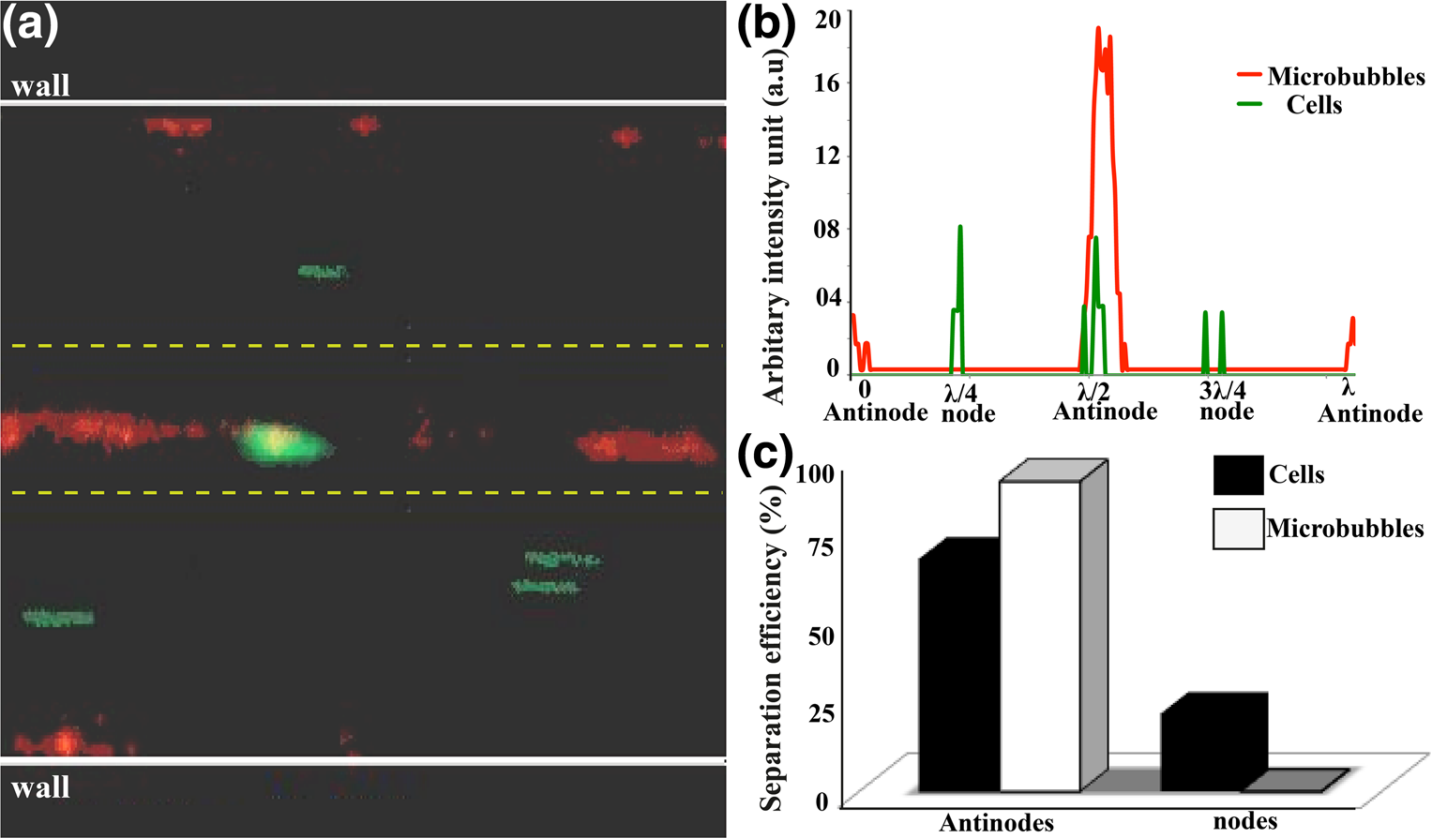
MBs assisted cell sorting in flow condition. **a** Merged image of frames shows the MBs (*red streaks*) and Mb-cell complex (*red-green overlapping streaks*) flowing through the centre of the capillary via antinodes under acoustics and cells (as *green streaks*) passing through nodes. **b** The fluorescent intensity peaks representing lateral distribution of MB (*red*), cells (*green*) and MB-cell complex (*red-green overlap*) at nodes and antinodes. **c** Sorting efficiency of 75% of MBs-cell complex at flow rate of 180 μl/min. 100% of the MBs go to antinode

Finally, we introduced the immuno-affinity captured cancer cells together with the unbounded cells at a flow rate of 180 μl/h and at driving frequency of 2.8 MHz corresponding to an acoustic pressure of 60 kPa. In Fig. 5a the merged image of cells and MBs-cell complex. The cells and MBs were flowing through the nodes and antinodes, respectively. Since we used a single inlet and single outlet chip, we made continuous recordings and calculated the efficiency of bounded cell separation to antinode. Figure 5b describes the lateral distribution of cells and MBs, MB-cell complex with green and red, red-green overlapping fluorescent peaks. Figure 5c is showing that cell-MB complexes are sorted with 75% efficiency. It is to be noted that some of the MB-cell complex are lost to the walls and not counted. Furthermore, 100% of the MBs migrate to the antinode and we could not observe any MB-cell complex at the nodes. These observations indicate that the BAACS is extremely efficient in sorting positive cells from background cells. Future design of the microfluidic chip will include two inlet and two outlet design to include sheath flow from the side walls to avoid MB-cell capture and efficient separation of sorted cells.

The strong negative contrast property of MBs, coupled with acoustofluidics has the potential to become a critical enabling technology for the development of cell sorting systems. Shi et al. have used buoyancy based separation of rare tumor cell by selectively binding them with anti EpCAM to Perfluorocarbon gas-filled MBs (Shi et al. 2013). Similarly Liou et al. (2015) have recently shown buoyancy activated cell sorting using Albumin MBs. Buoyancy based approach has also been shown earlier by Hsu et al. (2015), where CD4 + T cell were isolated by attaching them selectively to glass bubbles. Instead of using buoyancy-based approach where floating isolated cells are collected we utilize the acoustical properties of MBs to perform cell sorting. Such approach provides higher degree of control in positioning and isolating cells of interest with a continuous flow through conditions. Hence, sorting of MBs conjugated with cells should warrant the development of microfluidic lab-on-chip platforms for various sample preparation and analysis platforms. Finally, it is worth to reiterate that acoustophoresis in microfluidic systems is a mature technology, commonly performed in well-defined acoustic standing wave resonators to enable controlled migration/transport of cells. As such, the developed MB-activated acoustic cell sorting (BAACS) adds important selection criteria and has a potential to replace bulky and expensive FACS systems at the point of care.

## Conclusion

We report here an acoustics based cell separation method that relies on the strong negative contrast property of microbubbles to continuously separate cells. We term this method “microBubble-Activated Acoustic Cell Sorting (BAACS)”, and rely on target cells that are conjugated with MBs upfront with specific antibodies on their surface for continuous cell separation using ultrasonic standing wave. We have successfully developed and demonstrated the BAACS method with cell sorting efficiency of more than 75%. Notably is that all the cells conjugated with bubble migrate towards the antinode while the unbound cells migrate to the nodes. As a proof of principle the sorting is performed using a single inlet and single outlet device. Hence, once an optimized device has been developed, we expect the system to have higher efficiency. Hence, the method can be further developed as an alternative to FACS as an miniaturized cost effective cell sorter for point of care testing (POCT).

## Electronic supplementary material

The MBs can be sorted in flow through conditions in standing waves, where they are flowing at the antinode at the center of the channel.

Supplementary video 1 The bubbles are accumulated at the antinode at the middle of the channel and sorted at optimum flow rate and increasing the acoustic radiation pressure by optimizing the voltage (Vpp). (MP4 27,781 kb)

Supplementary video 2 The acoustic radiation pressure is increased beyond the optimum condition, resulting in MBs accumulation and migration to antinodes along the walls. (MP4 4956 kb)
